# Phase I/II clinical trial of enzyme replacement therapy with idursulfase beta in patients with mucopolysaccharidosis II (Hunter Syndrome)

**DOI:** 10.1186/1750-1172-8-42

**Published:** 2013-03-18

**Authors:** Young Bae Sohn, Sung Yoon Cho, Sung Won Park, Su Jin Kim, Ah-Ra Ko, Eun-Kyung Kwon, Sun Ju Han, Dong-Kyu Jin

**Affiliations:** 1 Ajou University Hospital, Ajou University School of Medicine, Suwon, Korea; 2 Department of Pediatrics, Samsung Medical Center, Sungkyunkwan University School of Medicine, 50 Irwon-dong, Seoul, Gangnam-ku 135-710, South Korea; 3 Department of Pediatrics, College of Medicine, Kwandong University, Cheil General Hospital & Woman’s Health Care Center, Seoul, South Korea; 4 Department of Pediatrics, Myongji Hospital, Kwandong University College of Medicine, Goyang, Korea; 5 Clinical Research Center, Samsung Biomedical Research Institute, Seoul, Korea

**Keywords:** Mucopolysaccharidosis II, Hunter syndrome, ERT, Recombinant iduornate-2-sulfatase, Idursulfase beta

## Abstract

**Background:**

Mucopolysaccharidosis II (MPS II, Hunter syndrome) is a rare X-linked lysosomal storage disorder caused by the deficiency of iduronate-2-sulfatase (IDS). In affected patients, glycosaminoglycan (GAG) accumulates in the lysosomes of many organs and tissues contributing to the pathology associated with MPS II. The objective of this phase I/II clinical study was to evaluate the efficacy and safety of recombinant human iduronate-2-sulfatase (idursulfase beta, Hunterase^®^) in the treatment of MPS II.

**Methods:**

Thirty-one MPS II patients between 6 and 35 years of age were enrolled in a randomized, single-blinded, active comparator-controlled phase I/II trial for 24 weeks. Patients were randomized to active comparator infusions (N=11), 0.5 mg/kg idursulfase beta infusions (N=10), or 1.0 mg/kg idursulfase beta infusions (N=10). The primary efficacy variable was the level of urinary GAG excretion. The secondary variables were changes in the distance walked in 6 minutes (6-minute walk test, 6MWT), echocardiographic findings, pulmonary function tests, and joint mobility.

**Results:**

Patients in all three groups exhibited reduction in urine GAG and this reduced GAG level was maintained for 24 weeks. Urine GAG was also significantly reduced in the 0.5 mg/kg and 1.0 mg/kg idursulfase beta groups when compared to the active comparator group (*P* = 0.043, 0.002, respectively). Changes in 6MWT were significantly greater in the 0.5 mg/kg and 1.0 mg/kg idursulfase groups than in the active comparator group (p= 0.003, 0.015, respectively). Both idursulfase beta infusions were generally safe and well tolerated, and elicited no serious adverse drug reactions. The most frequent adverse events were urticaria and skin rash, which were easily controlled with administration of antihistamines.

**Conclusions:**

This study indicates that idursulfase beta generates clinically significant reduction of urinary GAG, improvements in endurance as measured by 6MWT, and it has an acceptable safety profile for the treatment of MPS II.

**Trial registration:**

ClinicalTrials.gov: NCT01301898

## Background

Mucopolysaccharidosis II (MPS II, Hunter syndrome) is a rare X-linked lysosomal storage disorder caused by a deficiency of iduronate-2-sulfatase (IDS), involving in the catabolism of glycosaminoglycans (GAG) [1]. Mutations in the *IDS* gene located at Xq28 are responsible for MPS II [2-5]. Affected patients show a progressive accumulation of GAG in the lysosomes of many organs and tissues, which contribute to the clinical manifestations of MPS II. The common symptoms and signs include developmental delay, coarse face, short stature, skeletal abnormalities (dysostosis multiplex), joint contracture, hepatosplenomegaly, upper airway obstruction, and valvular heart disease. The phenotypical spectrum of MPS II is variable: In patients with the severe form, the onset of clinical symptoms is usually between 2 and 4 years of age, with progressive neurologic symptoms that lead to cognitive impairment [1]. Patients die in the first or second decade, usually due to obstructive airway disease and/or cardiac failure associated with progressive neurologic deterioration [1]. On the other hand, patients with the attenuated form show milder somatic symptoms and minimal neurological involvement. These patients have normal intelligence and survive into adulthood [6].

The treatment of MPS II was palliative prior to the introduction of enzyme replacement therapy (ERT). Unlike favorable result of hematopoietic stem cell transplant (HSCT) in MPS I [7], several attempts of HSCT for the treatment of MPS II showed unfavorable results [8]. In contrast, successful clinical trials [9,10] have led to the approval of ERT with human recombinant idursulfase (Elaprase^®^, Shire Human Genetic Therapies, Lexington, MA) by the United States Food and Drug Administration (FDA) and this therapy has been available since 2009 in Korea.

Pre-clinical studies using a knockout mouse model of MPS II suggested that 24 weeks of ERT with idursulfase beta (Hunterase^®^, Green Cross Corp., Yongin, Korea) was effective in reducing urinary GAG excretion and stored GAGs in several tissues, including liver, spleen, heart, lung, and kidney (unpublished data). In this report, we describe the results of a 24-week randomized, single-blinded, active comparator-controlled, phase I/II clinical trial of idursulfase beta designed to evaluate its efficacy and safety in the treatment of MPS II patients.

## Methods

### Patients

Thirty-one male patients between 6 and 35 years of age who were clinically and biochemically confirmed MPS II were eligible for enrollment. The clinical criteria included having a typical clinical disease compatible with MPS II, such as coarse facial features, hepatosplenomegaly, and dysostosis multiplex. The biochemical criteria were defined as an IDS activity < 5% of normal in leukocytes or fibroblasts. Patients who had undergone tracheostomy or previous bone marrow transplantation were excluded. All adult patients or the parents or guardians of patients under 18 years old provided written informed consent prior to enrollment. All patients were treated with the 0.5 mg/kg/week dose of active comparator (idursulfase; Elaprase^®^, Shire Human Genetic Therapies, Lexington, MA) for a mean duration of 15±0.9 months.

### Study design

This study was a 24-week randomized, single-blinded, active comparator-controlled clinical. The study was approved by the Institutional Review Board of the Samsung Medical Center, Seoul, Korea.

The 31 patients were randomized into one of three treatment arms: a comparator group, 0.5 mg/kg/week; an idursulfase beta group, 0.5 mg/kg/week; and an idursulfase beta group, 1.0 mg/kg/week. The three treatment arms were stratified based on age (under 12 years, between 12 and 18 years, and 18 years or older) and disease severity (severe and attenuated type). Once a patient enrolled in the study, previous Idursulfase (the active comparator) infusion was ceased 2 weeks prior to test drug infusion (the washout period). After the washout period, the study started with the 0.5 mg/kg/week dose of idursulfase beta arm and 0.5 mg/kg/week comparator arm (Step I). After 3 infusion doses of step I, the idursulfase beta 1.0 mg/kg/week arm started to get infusion after the safety was confirmed by the Safety Monitoring Board (Step II). All study infusions were performed in the Department of Pediatrics, Samsung Medical Center. The appropriate drug dosage was diluted with normal saline to yield a volume of 100 mL. The infusion rate was started at 8 mL/h and increased stepwise every 15 minutes, as follows: 16, 24, 32, and 40 mL/h. Patients were closely monitored during each infusion. If a patient developed any infusion reaction, including chills, fever, headache, flushing, or skin rash, the infusion was paused and medical intervention provided as needed. For subsequent infusions, patients were pre-medicated with antihistamines and/or steroids, depending on the severity of the symptoms. The duration of all subsequent infusions was lengthened, with a lower infusion rate.

After completion of 24 week study period, all of the patients restarted ERT with 0.5 mg/kg/week dose of idursulfase.

### Idursulfase beta

Idursulfase beta (Hunterase^®^, Green Cross Corp.,Yongin, Korea) is a recombinant protein that is produced using genetic engineering in CHO (Chinese Hamster Ovary) cell line. The idursulfase beta-producing cell line was generated by transfecting CHO DG 44 with an expression plasmid encoding the 550 amino acids of human iduronate-2-sulfatase. The idursulfase beta is secreted after 25 amino acid signal sequence is cleaved.

The idursulfase beta is produced by serum-free medium and suspension cell culture technology and is purified by several chromatography steps. The purity of purified protein is > 99.9% when analyzed by a number of chromatographic and electrophoretic methods.

### Efficacy assessments

The primary endpoint was the extent of reduction in urinary GAG excretion. Urine samples for GAG analysis were collected prior to each drug infusion, every 4 weeks from baseline. Urinary GAG concentrations were quantified by cerylpyridinium chloride (CPC) precipitation, normalized to urinary creatinine concentration, and reported as CPC unit/g creatinine.

The secondary endpoints included a 6-minute walk test (6MWT), forced vital capacity (FVC), heart size and function presented by left ventricular mass index (LVMI) and left ventricular ejection fraction (LVEF), and joint range of motions. The measurements of secondary variables were made at baseline and at Weeks 12 and 24.

For the 6MWT, subjects were instructed to walk forward and an observer recorded the total distance covered in 6 min, according to American Thoracic Society guidelines [11]. Two tests, typically one day apart, were performed during each evaluation and the farthest distance was used as the result for all analyses. The 6MWTs were conducted by a single specialist. Pulmonary function was assessed by spirometry to measure forced vital capacity (FVC). Two tests, typically one day apart, were performed during each evaluation and the largest values were used as the result for all analyses.

Heart size and function were assessed by an experienced pediatric cardiologist using a commercially available echocardiograph (Vivid 7, General Electric, 3.5-MHz transducer, Milwaukee, WI., USA) at the Department of Pediatrics, Samsung Medical Center. The left ventricular mass (LVM) was calculated based on 2D-guided M-mode echocardiographic measurements of the left ventricle. Measurements of the left ventricle internal dimension, interventricular septal thickness, and posterior wall thickness were obtained during diastole, according to the methods established by the American Society of Echocardiography. The LVM was calculated using the Devereux equation [12]. The LVM index (LVMI) was calculated by dividing the LVM by the height^2.7^, in order to minimize the effects of age, gender, ethnicity, and an overweight status [13-15]. Left ventricular hypertrophy (LVH) was defined as a LVMI >51 g/m^2.7^, a value greater than the pediatric 99th percentile associated with a 4.1-fold risk of cardiovascular morbidity in hypertensive adults [14,15].

Passive joint mobility was defined as the range of motion of the shoulder, elbow, wrist, hip, knee, and ankle joints, as assessed by one expert physician using universal goniometry method [16].

### Safety evaluations

Safety evaluations were performed at every visit; these included a physical examination, as well as measurement of vital signs, height, and weight. Adverse events (AE) were monitored and recorded throughout the study and assessed based on CTCAE (Common Terminology Criteria for Adverse Events) version 3.0 and classified by SOC/PT (System Organ Class/Preferred Terms) according to MedDRA (Medical Dictionary for Regulatory Activities) version 14.0. An adverse drug reaction (ADR) was defined as a responsible event occurring in association with drug infusion and the relationship of the drug infusion was judged by the investigator.

Immunogenicity tests consisted of screening for IgG antibodies to idursulfase beta at baseline, 12, and 24 weeks in all 31 Patients. Anti-idursulfase beta antibodies in plasma were detected by an enzyme-linked immunosorbent assay (ELISA) and positive results were confirmed by immunoprecipitation assays. If the initial screening was positive, the titer was determined. The assay sensitivity was approximately 200 ng/ml antibodies for ELISA. Neutralizing antibodies were detected by inhibition of cleavage of a fluorogenic substrate. Enzymatic activity neutralizing antibodies were detected using a potency assay for anti-idursulfase beta antibody positive samples.

### Pharmacokinetic study

The pharmacokinetic (PK) studies of idursulfase beta and the active comparator drug were performed in six patients (two patients from each group) at the ending of the study. Time points for blood sampling were immediately before dosing, and 1, 3, 4, 5, 6, 8, 12, 24 hrs post-dosing. The blood concentration of the drugs was measured by ELISA. Non-model interpretation of blood concentration curves was used to calculate the following PK parameters, if available in individual subjects, for interpretations with WinNonlin^®^ (version 5.2.1, Pharsight, USA): the area under the serum concentration (AUC) from the start of dosing to the last quantifiable time point (AUClast), the AUC from time zero to infinity (AUCinf), the apparent terminal half life (T1/2), the maximum observed peak plasma concentration (Cmax), and the time at which Cmax was observed (Tmax).

### Statistical analysis

Descriptive statistics were used for the other exploratory efficacy variables. All values were expressed as mean and standard deviation. Changes from baseline of urinary GAG excretion were evaluated using Analysis of Covariance (ANCOVA). Changes of secondary endpoints from baseline between the test drug and active comparator drug were investigated using the Student t-test. All analyses were performed with SAS, Version 9.2 (Cary, NC).

## Results

A total of 31 patients were screened and enrolled in the study. Patients were randomized into three treatment groups. Demographics and baseline characteristics of the patients are shown in Table 1. All patients were Korean males with MPS II. All 31 patients completed the full 24 weeks of treatment. All patients received all planned infusions.

**Table 1:** Patient demographic and baseline characteristics

	**Comparator 0.5 mg/kg/week (N=11)**	**Idursulfase beta 0.5 mg/kg/week (N=10)**	**Idursulfase beta 1.0 mg/kg/week (N=10)**
**Age, years**	10.8(±4.6)	11.7(±9)	11.5(±4.7)
**Height, cm**	124.7(±11.5)	119.6(±10)	122.2(±12.9)
**Weight, cm**	34.1(±9)	28.6(±7.8)	29.3(±8.9)
**Duration of previous ERT, month**	14.8(±0.8)	14.3(±0.6)	15.9(±0.7)
Urine GAG*, CPCunit/g Cr	129.1(±59.9)	164(±53.1)	124.7(±36.1)
**6MWT, m**	368(±76.8)	277.5(±100.2)	328(±70.1)
**FVC, L**	1.6(±0.5) (N=5)	1.5(±1) (N=3)	1.3(±0.4) (N=4)
**LVMI, g/m**^**2.7**^	55.6(±15.6)	59.5(±16.3)	54.4(±13.2)
**LVEF,%**	73.4(±6.7)	68.6(±3.8)	69(±4.1)

*reference range: < 175 CPC unit/g Cr for ≤ 9 years, < 85 CPC unit/g Cr for > 9 years.

### Primary efficacy outcome: urinary GAG excretion

**Figure 1:**
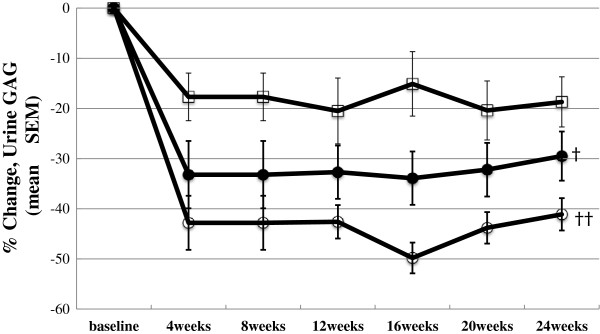
**Percent change in urinary GAG excretion in Korean male patients with Mucopolysaccharidosis II. **After 24 weeks of treatment, the percent changes in urine GAG levels were significantly greater in idursulfase beta groups treated at dosages of 0.5 mg/kg/week and 1.0 mg/kg/week, compared to the comparator group (†*P *= 0.043 and ††*P *= 0.002, respectively). Squares represent the comparator group (N = 11), closed circles represent the idursulfase beta 0.5 mg/kg/week group (N =10), and open circles represent the idursulfase beta 1.0 mg/kg/week group (N = 10).

The assessment results of urinary GAG levels are presented in Table 2. As shown in Figure 1, urine GAG level decreased in all three groups. Rapid decreases were observed in the first 4 weeks and the levels were maintained throughout the 24 weeks of treatment. Much more rapid declines were observed in the idursulfase beta groups than in the comparator group (Table 2, Figure 1). At 24 weeks of treatment, the percent changes of urine GAG levels were significantly greater in both the 0.5 and 1.0 mg/kg/week idursulfase beta groups than in the comparator group (-29.5±15.5 vs. -18.7±15.8, *P* = 0.043 and -41.1±10.2 vs. -18.7±15.8, *P* = 0.002, respectively). The percent changes between the two idursulfase beta groups were not significantly different (-29.5±15.5 vs. -41.1±10.2, *P* = 0.063).

**Table 2:** Urine GAG* excretion

	**Comparator 0.5 mg/kg/week (N = 11 )**	**Idursulfase beta 0.5 mg/kg/week (N = 10)**	**Idursulfase beta 1.0 mg/kg/week (N = 10 )**
**Baseline**	129.1(±59.9)	164(±53.1)	124.7(±36.1)
**4 weeks**	105.6(±56)	112.3(±56.7)	73.5(±35.7)
**8 weeks**	109.8(±67.8)	103.4(±56.4)	67.1(±21.7)
**12 weeks**	102.5(±55)	110.9(±44.1)	71.9(±24.4)
**16 weeks**	111.1(±69.7)	107(±46.2)	63(±24.6)
**20 weeks**	104.6(±62.6)	111.6(±46.5)	70.7(±24.5)
**24 weeks**	105.3(±59.6)	114.4(±45)	74.5(±27.3)

All values are menas ± SD.

*reference range: < 175 CPC unit/g Cr for ≤ 9 years, < 85 CPC unit/g Cr for > 9 years.

### Secondary efficacy outcome

The changes in secondary efficacy variables are presented in Table 3.

**Table 3:** Changes of secondary efficacy variables from baseline to 24 weeks

	**Comparator 0.5 mg/kg/week (N)**	**Idursulfase beta 0.5 mg/kg/week (N)**	**Idursulfase beta 1.0 mg/kg/week (N)**
**6MWT, m**	-9.1±35.2 (8)	61.6±32.2 (6)	38.4±36.4 (7)
**6MWT,% change**	-2.7±9.2 (8)	+23.5±16.9 (6)	+12.7±11.9 (7)
**FVC, L**	0±0.1 (5)	0±0.1 (3)	0.2±0.1 (4)
**FVC,% change**	+1.2±8.4 (5)	+7.9±11.3 (3)	+15.8±7 (4)
**LVMI,% change**	-1.7±18 (10)	-5.1±18.5 (10)	-5.9±25.1 (10)
**LVEF,% change**	+2.9±11.9 (9)	+2.5±10.8 (10)	-1.4±7.4 (10)
**Joint range of motion,% changes**			
**Shoulder flexion**	23.4±26.6 (10)	6.1±10.7 (10)	6.7±11.2 (10)
**Shoulder extension**	1.8±43 (10)	-20.4±31.8 (10)	-1.2±19.8(10)
**Elbow flexion**	7.2±10.7 (10)	1.1±9.4 (10)	-1.6±11 (10)
**Elbow extension**	25±78.8 (9)	3.8±41.6 (10)	11.9±94.5 (10)
**Hip flexion**	4.5±14 (10)	-4.1±14.9 (10)	-1.3±10.9 (10)
**Hip extension**	22.6±41.5 (10)	47.3±98.9 (10)	4.2±34.2 (8)
**Knee flexion**	4.5±7.9 (10)	-0.7±12.5 (10)	1.3±8.2 (10)
**Knee extension**	7.4±40.1 (9)	-28.1±59.1(10)	0±43.6 (8)

6MWT, 6 minute walk test.

FVC, functional vital capacity.

LVMI, left ventricular mass index.

LVEF, left ventricular ejection fraction.

#### 6 minute walk test

**Figure 2:**
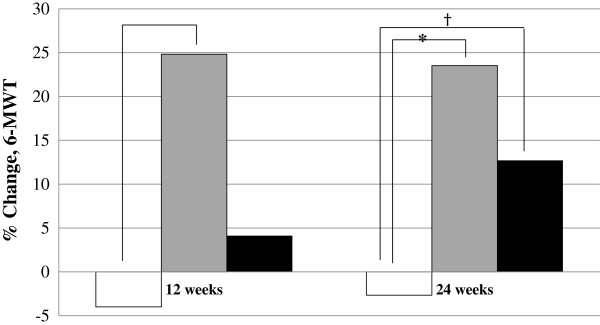
**Percent change in 6MWT distance in Korean male patients with attenuated type Mucopolysaccharidosis II. **At 24 weeks, the percent changes in 6MWT distance were significantly higher in idursulfase beta groups treated at dosages of 0.5 mg/kg/week (* P = 0.003) and 1.0 mg/kg/week († P = 0.015), compared to comparator group. White bars represent the comparator group (N = 8), grey bars represent the idursulfase beta 0.5mg/kg/week group (N = 6), and black bars represent the idursulfase beta 1.0mg/kg/week group (N = 7).

The 6MWT results of attenuated patients in each group were analyzed. The effect of treatment on the 6MWT distance during the study is shown in Figure 2. At the baseline, distance walked for 6 min was 293.1±93.1, 345.6±59.8, and 404.7±49.7 m in the idursulfase beta 0.5 mg/kg/week (N = 6), the idursulfase beta 1.0 mg/kg/week (N = 7), and the comparator group (N = 8), respectively. At 24 weeks, the percent change in the 6MWT distance was significantly increased in the idursulfase beta 0.5 mg/kg/week group (23.52±16.90 vs. -2.66±9.19, *P* = 0.003) and the idursulfase beta 1.0 mg/kg/week group (12.71±11.91 vs. -2.66±9.19, *P* = 0.015) compared to the comparator group (Figure 2).

#### Forced vital capacity

Obtaining subject cooperation for performing an exact pulmonary function test (PFT) was difficult; consequently, PFT was assessed in a small number of subjects in each group. At the baseline, the FVC was 1.5±1.0, 1.3±0.4 and 1.6±0.5 L in the idursulfase beta 0.5 mg/kg/week (N = 3), idursulfase beta 1.0 mg/kg/week (N = 4), and comparator (N = 5) groups, respectively. The FVC showed little change at 24 weeks compared with the baseline measurements in all three groups. The percent change in the FVC tended to be higher in the idursulfase beta 0.5 mg/kg/week (7.9±11.3 vs. 1.2±8.4, *P* = 0.40) and idursulfase beta 1.0 mg/kg/week groups, compared to the comparator (15.8±7 vs. 1.2±8.4 *P* = 0.10) group. However, no significant difference was noted among the three groups.

#### Cardiac evaluations

At the baseline, the mean LVMI was 59.5±16.3, 54.4±13.2 and 55.6±15.6, g/m^2.7^ in the idursulfase beta 0.5 mg/kg/week (N = 10), idursulfase beta 1.0 mg/kg/week (N = 10), and comparator (N = 10) groups, respectively. At 24 weeks of treatment, the LVMI of the patients decreased by 1.9 (5.1±18.5%), 0.6 (5.9±25.1%), and 0.9 (1.7±18%) g/m^2.7^ in the idursulfase beta 0.5 mg/kg, idursulfase beta 1.0 mg/kg, and comparator groups, respectively. However, none of these changes from baseline to 24 weeks was statistically significant. Comparisons among the study arms did not indicate any significant differences. The LV ejection fraction (EF) showed little change after 24 weeks of treatment in all three groups (Table 3). In addition, comparisons among the study arms showed no significant differences.

#### Joint range of motion

Changes in joint range of motion are shown in Table 3. Following 24 weeks of treatment, several joints had increased range of motion, including shoulder flexion, elbow extension, and hip extension. However, none of these changes resulted in significant improvements among the treatment groups.

### Safety evaluations

The 24-week treatments with idursulfase beta or comparator were well tolerated in the study population. The majority of AEs were considered to be unrelated to the study drugs and consistent with those expected in patients with MPS II. No serious AEs occurred during the study.

Table 4 presents the ADRs observed during the study period. Four cases occurred in 1/10 subjects (10%) in the idursulfase beta 0.5 mg/kg group, three cases occurred in 2/10 subjects (20%) in the idursulfase beta 1.0 mg/kg group, and 19 cases occurred in 2/11 subjects (18.6%) in the comparator group. Of these cases, urticaria (19 cases) was reported most frequently, followed by rash (4 cases), itching (2 cases), and wheezing (1 case). All of the ADRs were mild and easily controlled with an adjustment of the infusion rate or medications.

**Table 4:** Summary of adverse drug reactions

**Group**	**Number of subjects with events**	**Number of events**	**Events**
**Comparator 0.5 mg/kg/week (N = 11)**	2	19	Urticaria (13)
			Rash (4)
			Itching (1)
			Wheezing (1)
**Idursulfase beta 0.5 mg/kg/week (N = 10)**	1	4	Urticaria (3)
			Itching (1)
**Idursulfase beta 1.0 mg/kg/week (N = 10)**	2	3	Urticaria (3)

### Antibodies

Anti-idursulfase IgG antibodies were detected in 10 patients at baseline (comparator 0.5 mg/kg/week, 4; Idursulfase beta 0.5 mg/kg/week, 4; Idursulfase beta 1.0 mg/kg/week, 2). These antibodies might be a result of the previous idursulfase treatment, because all subjects had been treated with idursulfase for a median of 15 months before the clinical trial. At 12 and 24 weeks, the results of antibody screening tests were same as those from the baseline study. Therefore, no newly detected antibodies were evident and no antibodies had disappeared. No anti-idursulfase IgE antibodies were detected in any of the three groups at any time.

### Pharmacokinetic study

**Figure 3:**
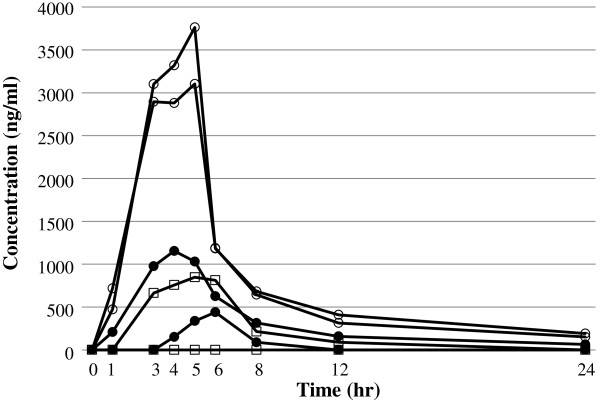
Plasma concentration of test drugs in Korean male patients with Mucopolysaccharidosis II Squares represent the comparator at 0.5 mg/kg/week group (N = 2), closed circles represent the idursulfase beta 0.5 mg/kg/week group (N = 2), and open circles represent the idursulfase beta 1.0 mg/kg/week group (N = 2).

The number of subjects was small in each group and their individual differences were considerable; however, an apparent biexponential elimination curve was observed when time courses of plasma drug concentrations were plotted (Figure 3). The idursulfase beta 0.5 mg/kg and 1.0 mg/kg groups (2 patients each) showed an apparent terminal half life of 7.3 and 9.1 hours, Cmax of 1024.9 and 2045.2 ng/ml, and AUClast of 2724 and 7804 ng h/ml, respectively. The comparator group showed a serum concentration under the detection limit for one patient and these data were not included in the analysis. In the other patient, the terminal half life was 2.0 hours, Cmax was 848.2 ng/ml, and AUClast was 4810 ng h/ml. The small number of subjects made statistical analysis between the groups impossible.

## Discussion

This study is the first active comparator controlled clinical trial of idursulfase beta for Korean male patients with MPS II. A dosage of idursulfase beta of 0.5 mg/kg/week had a significant effect on the primary endpoint, urinary GAG excretion, when compared to the active comparator. Idursulfase beta treatment also resulted in significant improvements in 6MWT distances in attenuated MPS II patients. Idursulfase beta showed similar effectiveness to the comparator in terms of other secondary efficacy variables, including FVC, echocardiography, and joint range of motion. A treatment dose of 0.5 mg/kg/week of idursulfase beta appeared to be sufficient, as the urinary GAG obtained with 1.0 mg/kg/week of idursulfase beta was not significantly different (Figure 1).

The safety profile of idursulfase beta, based on the frequency and severity of infusion related AEs, indicated that it was well tolerated and AEs were easily controlled by temporary pausing of infusion or antihistamine or steroid medications.

Despite the promising results, this study has several limitations. First, all of the subjects had been treated with idursulfase during the previous median 15 months. Although all 31 patients had a 2-week washout period, the consequences of previous treatment with 0.5 mg/kg/week of idursulfase need to be carefully considered. The efficacy of idursulfase was likely underestimated since some patients had neutralizing antibodies to idursulfase already formed as a result of the previous treatments. Therefore, a clinical trial of idursulfase beta with patients who have never been treated with ERT should be conducted. Secondly, the results of the PK study should be interpreted carefully due to the small sample size: only two subjects per group consented to and participated in the PK study. In addition, one subject in the comparator group had unreliable data; that patient was excluded from the analysis because the drug was undetectable in all of the PK samples. Furthermore, the fact that ELISA operates based on antigen-antibody reactions for analysis raises the possibility that this method may not be optimal for direct comparisons of serum concentration of the drugs. Because the subject who had undetectable serum concentration of the test drug was the only patient having neutralizing antibody among the six patients included in the PK study, the presence of neutralizing antibody could influence the ELISA assay.

## Conclusions

The idursulfase beta treatment was well tolerated in Korean patients with MPS II and resulted in a significant reduction in urinary GAG excretion and an improvement in the 6MWT distance when compared to the active comparator. The effectiveness in pulmonary function, cardiac function and joint mobility was similar to that of the active comparator. Further experiments will be needed, especially in patients who have never been treated with enzyme replacement, in order to establish the long-term efficacy and safety of idursulfase beta for the treatment of MPS II.

## Competing interests

The authors have no conflicts of interests.

## Authors’ contributions

Y.B. Sohn contributed to the research design, data analysis and interpretation, and drafting of paper. S-Y Cho, S.W. Park, S.J. Kim, and A-R Ko contributed to data analysis & interpretation, and critical review of the paper. E-k Kwon and S.J. Han participated in the acquisition of data. D.-K. Jin contributed to the research design, data analysis & interpretation, drafting and critical review of paper, and approval of the submitted paper. All authors have read and approved the final manuscript.
